# Personalized preictal EEG pattern characterization: do timing and localization matter?

**DOI:** 10.3389/fnins.2025.1526963

**Published:** 2025-05-02

**Authors:** Galya Segal, Noam Keidar, Moshe Herskovitz, Yael Yaniv

**Affiliations:** ^1^Laboratory of Bioelectric and Bioenergetic Systems, Faculty of Biomedical Engineering, Technion-Israel Institute of Technology, Haifa, Israel; ^2^Faculty of Medicine, Technion-Israel Institute of Technology, Haifa, Israel; ^3^Department of Neurology, Rambam Health Care Campus, Haifa, Israel

**Keywords:** EEG, epilepsy, preictal patterns, spectral entropy, Hjorth mobility

## Abstract

**Objectives:**

Better understanding of ictogenesis may allow clinical interventions and potentially reduce the impact of epilepsy on patients’ quality of life. This study aims to characterize the EEG changes during the preictal period.

**Methods:**

This work retrospectively analyzed long-term scalp EEG recordings collected at two neurology centers to characterize preictal activity (start point and duration) for each seizure using EEG features. A channel selection algorithm was implemented and localized preictal activity.

**Results:**

Out of 19 patients, 17 (89.5%) had a distinct preictal pattern, starting 83 ± 60 min before seizure onset and lasting 56 ± 47 min. Spectral Entropy and Hjorth mobility were consistently two out of the three features best distinguished preictal from interictal activity. The third distinguishing feature was either theta power, delta power, beta power, or gamma power. Preictal activity before two seizures in the same patient shared common electrodes and features but differed in duration and timing.

**Conclusion:**

Preictal activity, defined as prolonged intervals of uncommon EEG activity, varies in time, localization and signal patterns between individuals and varies in timing and duration between seizures of the same individual.

## 1 Introduction

Epilepsy is a neurological disease characterized by pathological synchronized neuronal activity known as seizures (Lowenstein, 2018). It is estimated that 50 million people worldwide suffer from epilepsy (WHO, 2019), with 30% of whom being resistant to medications (Kalilani et al., 2018). Given the abrupt and unpredictable onset of seizures, epilepsy has a range of cognitive, psychological and social ramifications, which significantly impact quality of life (Mormann et al., 2006). Patients with epilepsy are also at increased risk of falls which can lead to severe injuries (Kalilani et al., 2018). A comprehensive understanding of the pathophysiological mechanisms underlying seizures is crucial for identification of the preictal phase, i.e., the period before seizure onset. Better characterization of preictal activity will enable both prediction of imminent seizures and timely clinical intervention, such as fast-acting anticonvulsant drugs or vagal nerve stimulation (Mormann et al., 2006), thereby reducing the impact of epilepsy on patients’ quality of life.

Over the years, various studies have attempted to identify preictal patterns in electroencephalography (EEG) signals. Early works used linear measures in the time and frequency domains of intracranial EEG (iEEG) signals to identify patterns in the preictal period several seconds before seizures onset (Viglione and Walsh, 1975). Later works delineated numerous measures for the preictal state, such as the entropy of the signal, which quantifies the complexity and irregularity of the signal (Rogowski et al., 1981; Martinerie et al., 1998). However, the quality of these measures has been questioned (Mormann et al., 2006), due to poor performance on larger databases. Additionally, early studies were limited to the preictal period without considering the interictal period, which is the seizure-free interval outside the presumed preictal period (Mormann et al., 2006), resulting in low specificity. Importantly, many studies were conducted on iEEG data, which require an invasive procedure which can be both hazardous to patients and challenging for clinical trials. This underscores the necessity for studies based on long-term scalp EEG recordings that include interictal periods for control.

More recent work used iEEG recordings and short (∼1 h long) scalp EEG recordings to develop and train sophisticated models to distinguish between preictal and interictal periods (Truong et al., 2018; Tsiouris et al., 2018; Abdelhameed and Bayoumi, 2019; Duy Truong et al., 2019; Rasheed et al., 2020; Wang et al., 2020). Despite advances in model performance, few systems were tested in clinical trials. This can be explained by the need for physiological understanding of seizure initiation. In their review of advances in seizure prediction tools, Kuhlmann et al. (2018) emphasized the importance of a neurophysiological understanding of the preictal state and the need for long-term recordings for accurate prediction.

Another explanation for the failure of prediction models is the prior assumption that preictal period onset and duration are identical for all patients. Therefore, a personalized approach might be more suitable.

The present study proposes a novel means of personalized characterization of the preictal period, and uses it to inspect the timing, duration, localization and EEG patterns of the preictal period. Long-term scalp EEG recordings from two neurology centers were retrospectively analyzed to characterize the preictal period in each record using features common in the field of neuroscience. Simple machine learning models were also used to identify the features that most contribute to personalized preictal characterization and generate a method for feature importance. In addition, data regarding the electrodes in which preictal activity was most prominent in each patient were leveraged to develop a channel selection method.

## 2 Materials and methods

### 2.1 Data

The initial database included 120 scalp EEG recordings from 47 patients acquired at the Department of Neurology at Rambam Health Care Campus in Israel (ethics number: 0833-20-RMB), or the Neurology Department of Siena University Hospital in Italy (Detti et al., 2020; Detti Paolo, 2020). The recordings from the latter site were accessed from the Siena Scalp EEG Database. Seizure onset zone and time onset were determined by a neurologist specializing in epilepsy after a careful review of the clinical and electrophysiological data of each patient for both Rambam and Siena datasets.

Some patients had a single seizure recorded, while others had multiple seizures recorded. To avoid interference from post-ictal effects and focus on the transition from stable preictal state to seizure onset (Liang et al., 2020; Kudlacek et al., 2021; Sumsky and Greenfield, 2022; Bower, 2024; Pedersen et al., 2024), in case a cluster of seizures was recorded, only the first seizure in the cluster was analyzed. However, seizures recorded at least 12 h apart from each other were not considered from the same cluster. And therefore, were analyzed as separate seizures.

**FIGURE 1:**
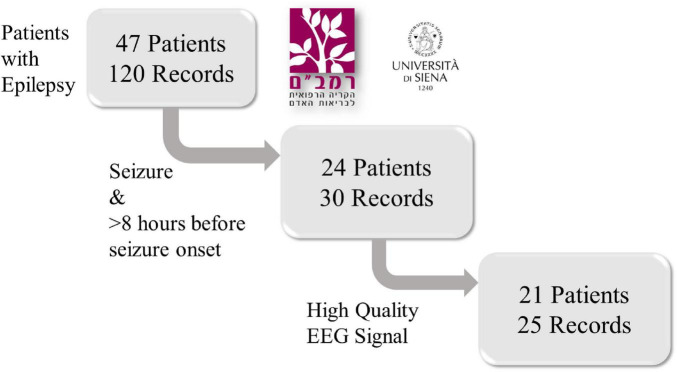
A flowchart of the retrospective study design. Scalp EEG recordings from 47 patients with epilepsy were collected from the Rambam Health Care Campus in Israel and the Sienna University Hospital in Italy. Only records containing at least one epileptic seizure and at least 8 h of signal prior to seizure onset were included. Then, five recordings were removed due to low signal quality and high noise levels. After exclusion, 25 records from 21 patients were included.

Recordings with more than 10% of their time had values exceeding the transducer’s maximum value were classified as noisy and removed from the dataset. Consequently, five recordings were excluded due to low signal quality and high noise levels. The final analysis was performed on 25 records from 21 patients (Figure 1), representing 644 h of EEG and 54 seizures. Gender, age and epilepsy type of each patient in the inclusion criteria and information regarding intracranial monitoring or resection surgery are shown in Table 1.

**TABLE 1:** Patient data.

Patient ID	Gender	Age	Epilepsy	Seizure onset zone	Duration of recordings (h:min:s)	Number of seizures	Intracranial monitoring or resections
**Sienna University Hospital**							
4	M	37	Left temporal	Unknown	13:29:16	2	Unknown
**Rambam Health Care Campus**							
1	F	24	Right and left temporal	F8 T4 T6	23:59:47	2	No
3	M	57	Left parietal-operculum	F8 F7 T4 T3	23:59:43	1	No
4	F	40	Right temporal	F8	11:26:41	1	No
5	F	36	Right temporal	F8	47:59:28	3	Yes
7	M	52	Right temporal	Fp2	49:26:13	2	No
8	F	35	Left frontal	Fp1	08:48:47	1	Yes
11	F	28	Right temporal	F8	10:03:59	1	No
12	M	18	Right temporal	F4 C4	14:56:53	1	No
13	M	32	Left temporal	Fp1 F3	23:59:51	1	Yes
14	F	22	Left insular	F7	11:58:03	1	No
17	F	18	Left anterior temporal	F7	36:33:32	3	Yes
18	F	18	Right Parieto-occipital	P4 O2	96:35:53	6	Yes
19	F	41	Left temporal	Fp1 F3	21:25:10	2	No
23	F	23	Left temporal	F7	23:59:45	4	No
25	F	35	Right parietal	Fp1 F3 Fp2 F4	40:04:58	3	Yes
26	F	28	Left temporal	F7	44:55:14	3	No
28	F	34	Left temporal	F7	71:59:46	8	No
29	F	19	Right frontal	Fp2 F4	23:54:04	3	No
31	M	23	Left hemisphere	P3 T3 T5	47:59:45	4	Yes
32	M	24	Left occipito-temporal	T3 T5	45:36:34	2	Yes

Gender, age, epilepsy type, total duration of records and number of seizures included in the records are shown for each patient in the inclusion criteria. Patient 5 had a right frontotemporal lobectomy due to a type 2 dysplasia. Patient 8 had a resection. Patient 13 had a left frontal tumor. Patient 17 had a resection of a lesion in her left temporal lobe, at the uncus, which was found to be a ganglioglioma. Patient 18 had a right occipitoparietal cortical dysplasia. She had a resection surgery and currently she has hemianopsia and is also seizure-free. Patient 25 had an intracranial monitoring and her epileptic focus was found to originate from her inferior parietal lobe. Patient 31 had hemispherectomy, and is currently seizure free. Patient 32 had intracranial monitoring with and his epileptic focus was found to originate from a left tempo-occipital location.

The electrodes were arranged according to the international 10–20 system. Signal sampling frequency was 256 Hz for the Rambam database and 512 Hz for the Siena database.

Each recorded seizure was annotated for start time and end time by neurologists specializing in epilepsy.

### 2.2 Verification by a specialist physician

All recordings from the Rambam dataset in which the preictal interval was detected by the algorithm described in the following section were manually reviewed by a neurologist specializing in epilepsy. Both EEG signals and video recording were examined to confirm that preictal activity detected was not explained by artifacts or any other clinical activity such as sleeping, eating, talking, etc.

### 2.3 Preprocessing

The raw EEG signal contained constant trend and high-frequency noise due to patient movement and acquisition noise. Therefore, frequencies below 0.5 Hz and above 75 Hz were filtered with a digital finite impulse response bandpass filter. Preprocessing, feature extraction and data analysis described in the methods were implemented using Python (Guido and Drake, 1995), NumPy (Harris et al., 2020), scikit-learn (Buitinck et al., 2013), Matplotlib (Hunter, 2007), eeglib (Cabañero-Gomez et al., 2021), EntropyHub (Flood and Grimm, 2021), and PyEEG (Bao et al., 2011).

### 2.4 Feature extraction

Ten features were extracted from each channel using a moving window analysis to achieve an efficient representation of the signal (Mormann et al., 2006). All features (see below) are widely used in neuroscience and have been proven to be important in the field of seizure prediction and detection (Mormann et al., 2006; Truong et al., 2018; Segal et al., 2023). Based on previous research, the optimal window size ranges from 4 to 10 s (Hjorth, 1970; Mormann et al., 2005; Hoyos-Osorio et al., 2016). Larger windows, for example above 1 min, summarize a long period of time and are insensitive to frequent changes in the EEG; it may even summarize the begging, duration and ending of an entire seizure. Too small window captures frequent changes in the EEG but may result in poor representations of measurements that require a sufficient number of samples such as Detrended Fluctuation Analysis (Hardstone et al., 2012) or band frequencies. Therefore, an intermediate-size window of 6 s was chosen. Each feature was calculated over 6 s windows, with an overlapping window of 3 s. The following section provides a detailed description of each feature (Equations 1–13).

### 2.5 Features

#### 2.5.1 Spectral entropy

The spectral entropy is a measure of the spectral power distribution that quantifies the irregularity or complexity of a signal in the frequency domain (Shannon, 1948; Inouye et al., 1991; Kannathal et al., 2005).

Let *x*(*t*) be a signal in time, and *x*(*n*) be the discrete sampled signal. The power spectrum of the signal is *S* (*m*) = |*X* (*m*)|^2^ where *X* (*m*) is the discrete Fourier Transform of *x* (*n*), M is the length of the discrete Fourier Transform, and *m* = 0, 1, …*M* − 1. The probability distribution of *S* (*m*) is described as:


(1) $$P\,\left(m\right)=\frac{S\,\left(m\right)}{\sum_{i=0}^{M-1}S\,\left(i\right)}$$

The spectral entropy *H* is described as the Shannon entropy of the spectral power distribution:


(2) $$H=-\sum_{m=1}^{M}P\,\left(m\right)\,l\,o\,g_{2}\,P\,\left(m\right)$$

Entropy (Shannon, 1948) can be interpreted as the average level of information or uncertainty in a random variable.

#### 2.5.2 Hjorth parameters

Hjorth parameters are characteristics of EEG in the time domain (Hjorth, 1970). The *Hjorth mobility* is the square root of the ratio between variances of the first derivate of the signal *x*(*n*) and the amplitude:


(3) $$M\,o\,b\,i\,l\,i\,t\,y=\sqrt{\frac{v\,a\,r\,\left(\frac{d\,x\,\left(n\right)}{d\,n}\right)}{v\,a\,r\,\left(x\,\left(n\right)\right)}}$$

It measures the standard deviation of the slope of the signal with reference to the standard deviation of the amplitude. It can also be conceived also as the mean frequency of the signal.

The *Hjorth complexity* is the ratio between the mobility of the first derivative of the signal and the mobility of the signal itself:


(4) $$C\,o\,m\,p\,l\,e\,x\,i\,t\,y=\frac{M\,o\,b\,i\,l\,i\,t\,y\,\left(\frac{d\,x\,\left(n\right)}{d\,n}\right)}{M\,o\,b\,i\,l\,i\,t\,y\,\left(x\,\left(n\right)\right)}$$

A signal is said to have minimal complexity if it has a discrete frequency in the spectrum, meaning it is a pure sine function in the time domain.

#### 2.5.3 Higuchi fractal dimension

A fractal is a shape that retains its structural detail in different scales. Complex objects can thus be described by fractal dimensions. The Higuchi fractal dimension (HFD) (Higuchi, 1988) originates from chaos theory and has been applied as a complexity measure of physiological signals such as EEG (Kesić and Spasić, 2016).

For a discrete-time signal *x* (*n*) consisting of *N* data points, a free parameter *k*_*max*_ ≥ 2, and *m* ∈ {1,2, …, *k*_*max*_}, the length *L*_*m*_(*k*) is defined by:


(5) $$L_{m}\,\left(k\right)=\frac{N-1}{\left\lfloor \frac{N-m}{k}\right\rfloor \,k^{2}}\,\sum_{i=1}^{\left\lfloor \frac{N-m}{k}\right\rfloor }\left|x\,\left(m+i\,k\right)-x\,\left(m+\left(i-1\right)\,k\right)\right|$$

The length *L*(*k*) is defined by the average value of the *k* lengths *L*_1_(*k*), *L*_2_(*k*), ….*L*_*k*_(*k*) as follows:


(6) $$L\,\left(k\right)=\frac{1}{k}\,\sum_{m=1}^{k}L_{m}\,\left(k\right)$$

When fitting a linear function through the data points $\left\{\left(l\,o\,g\,\frac{1}{k},l\,o\,g\,L\,\left(k\right)\right)\right\}$, the slope of the least square best fit is defined to be the HFD of *x*(*n*).

#### 2.5.4 Frequency band power

The band power is a fraction of the spectral energy of the signal in a given frequency interval. Given a signal *x*(*n*) with a discrete Fourier transform *X*(*m*) and power spectrum *S*(*m*), the band power between two frequencies *f*_1_, *f*_2_ where *f*_1_ < *f*_2_ is:


(7) $$B\,a\,n\,d\,P\,o\,w\,e\,r=\sum_{f=f_{1}}^{f_{2}}S\,\left(m\right)$$

The *Delta power* is the fraction of spectral energy in the delta band, which is the interval between 0.4 and 4 Hz. Mormann et al. (2005) showed a decrease in the delta power before seizure onset. The *Theta power* is the fraction of spectral energy between 4 and 8 Hz, the *Alpha power* is between 8 and 13 Hz, the *Beta power* is between 13 and 30 Hz and the *Gamma power* is between 30 and 48 Hz.

#### 2.5.5 Detrended fluctuation analysis

Detrended fluctuation analysis (DFA) (Peng et al., 1994) enables measurement of the self-similarity of an non-stationary signal. Self-similarity means that the statistical properties of a small part of the signal are scaled versions of the whole signal in the time dimension (Hardstone et al., 2012).

First, the signal *x*(*n*) is converted to a mean-centered cumulative sum:


(8) $$z_{t}=\sum_{i=1}^{t}\left(x\,\left(i\right)-x_{a\,v\,g}\right)$$

where $x_{a\,v\,g}=\frac{1}{N}\,\sum_{i=1}^{N}x\,\left(i\right)$ is the average of *x*(*n*). This version of the signal presents longer trends in the signal. Then, a set *T* = {*n*_1_, *n*_2_, …, *n*_*k*_} of integers is selected such that *n*_1_ < *n*_2_ < … < *n*_*k*_ ≤ *N*, and such that the sequence is distributed approximately evenly in log-scale. This set defines a log-spaced scale. For each *n*ε*T*, the cumulative sum *z_t_* is divided into consecutive segments *S*_1_, *S*_2_, …. *S*_*m*_ each of length *n*, where *m* is the number of segments for a given *n*. For each segment *S_i_*, a straight line is fitted and the least square error *e*_*i*_ is calculated. In other words, the root mean square deviation from the local trend is calculated for each segment. The root mean square of all *e_i_* for a given *n* is calculated:


(9) F⁢(n)=1m⁢∑i=1mei2

Repeating this process for all *n*ε*T* gives a root mean square value *F*(*n*) for each scale *n*. Then, a linear line is fitted between the log-*n* and log- *F*(*n*). The slope of this linear line is the DFA of the signal *x*(*n*).

### 2.6 Distance in feature-space

The *interictal interval* was defined as the time interval of 2–4 h, ending 6 h before seizure onset, and was taken as a representative of the interictal state. This time interval included *N* time points, each characterized by 10 features. The *N* time points enabled estimation of the distribution of the interictal (non-epileptic) state for each patient in a 10-dimension feature space.

The preictal period was located somewhere in the time interval between the end of the interictal interval and seizure onset. The distance between each time-point in that interval and the interictal distribution was measured using the Mahalanobis Distance:


(10) $$d\,\left(\vec{p},D\right)=\sqrt{\left(\vec{p}-\vec{\mu }\right)\,S^{-1}\,\left(\vec{p}-\vec{\mu }\right)}$$

which is a measure of the distance from a point $\vec{p}={\left(p_{1},p_{2},\ldots ,p_{M}\right)}^{T}$ and a sample distribution *D* on ℝ^*M*^ with mean $\vec{\mu }={\left(\mu_{1},\mu_{2},\ldots ,\mu_{M}\right)}^{T}$ and positive-definite covariance matrix *S*; *M* is the dimension of each time point. In the present use case, $\vec{p}$ in a 10×1 vector as each point in time is represented by 10 features, and *D* is a 10×*N* matrix representing the interictal distribution of *N* time points using 10features (Mahalanobis, 1936). This distance represents how far a time point is from interictal behavior. Our hypothesis is that preictal activity can be distinguished from interictal activity in feature space, with high distances from seizure onset suggesting preictal activity.

Let us define the following: *t_1_*, *t_L_* are start and end times of the preictal interval, respectively, *d_t_* is the Mahalanobis distance corresponding to a specific time *t* in that interval, so that *d*_50_ is the median of the Mahalanobis distances that interval and *L*_99_ is the 99^th^ percentile of the Mahalanobis distance of each interictal point from the sample distribution *D*.

The preictal interval was defined as follows:


(11) $$d_{50}>L_{99}$$

(12) $$t_{L}-t_{1}\geq 15\,m\,i\,n$$

Namely, the preictal interval is defined as a time interval that meets two requirements: first, most of the values in that interval exceed the 99^th^ percentile of the Mahalanobis distance of the interictal interval. Therefore, it is statistically distinct from the interictal distribution in feature space. Second, the duration of the preictal interval must be at least 15 min, so it is sufficiently long enough to differ from noise or artifacts.

Based on the definition above, preictal period was located and labeled in each record in the inclusion criteria.

### 2.7 Channel selection

For each seizure, all data points in the preictal interval and interictal interval preceding the seizure were randomly split into a train set and a test set, when the test set was 20% of the data. Then, a logistic regression model was trained on the test set and tested on the train set. The model was evaluated by the *F_1_* score, which is the harmonic mean of precision and recall, was calculated:


(13) $$F_{1}=2\,\frac{p\,r\,e\,c\,i\,s\,i\,o\,n\cdot r\,e\,c\,a\,l\,l}{p\,r\,e\,c\,i\,s\,i\,o\,n+r\,e\,c\,a\,l\,l}$$

The *F_1_* score consists of both precision and recall. Therefore, it is a good measure of the ability of the model to distinguish between the two populations and allows a simple and unambiguous ranking of the channels. This process was repeated for all channels, and each channel was rated by the *F_1_* score of the regression model fitted to that channel.

The *F_1_* score quantifies how well the preictal interval separates from the non-preictal interval in the 10-features space. Therefore, high *F_1_* score indicates that the channel is indicative of prominent preictal activity. The *F*_1_ scores were arranged by ascending order, and those above the 75*^th^* quantile were selected. The corresponding channels were selected as most indicative of preictal activity.

### 2.8 Feature importance

The 10 features represent different aspects of scalp EEG signals and contribute differently to the differentiation between preictal and non-preictal intervals. After the channel selection algorithm was applied, EEG channels were ranked based on the *F*_1_ scores of the logistic regression models fitted to each channel. The maximal *F*_1_ score was attributed to the channel most indicative of preictal activity. The weight coefficients of the logistic regression model fitted to that channel were then used to identify the features most important for this separation. The absolute value of the weight coefficient determined their order of importance.

**FIGURE 2:**
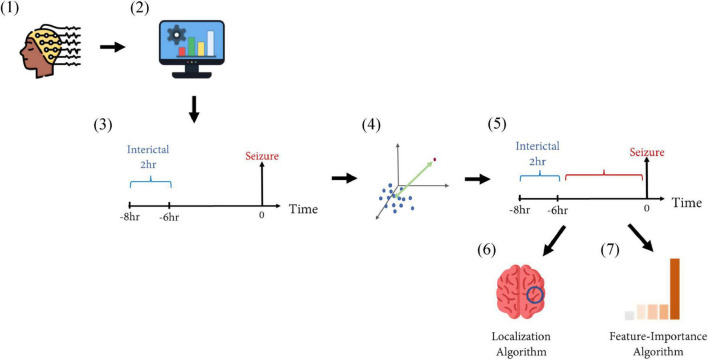
Schematic representation of the methods. (1) EEG recordings from patients with epilepsy were collected from two medical centers. (2) The recordings were filtered using a band-pass filter of 0.5–75 Hz. Then, 10 features were extracted from the filtered signals in a moving-window fashion. (3) The inter-ictal interval (marked in blue) was defined as a period of 2 h ending 6 h before seizure onset, representing the inter-ictal, non-epileptic distribution in a 10-feature space. (4) The distance between a time point to the inter-ictal distribution was calculated via the Mahalanobis distance. (5) This distance was calculated for each time point during the time interval of 6 h before seizure onset (marked in red). This was calculated for each recording, and prolonged intervals of high distance measures defined the preictal interval. (6) A Localization Algorithm selected the EEG electrodes showing maximal separation between pre ictal and inter-ictal intervals. (7) A Feature-Importance Algorithm selected the features contributing most to such selection.

After the preictal interval was determined, the Channel Selection algorithm was applied and channels showing best separation between preictal to interictal intervals were selected. Based on the selected channels, the Feature-Importance algorithm was applied and the top three features identified by this algorithm were selected for each seizure (Figure 2).

## 3 Results

### 3.1 Preictal interval definition

**FIGURE 3:**
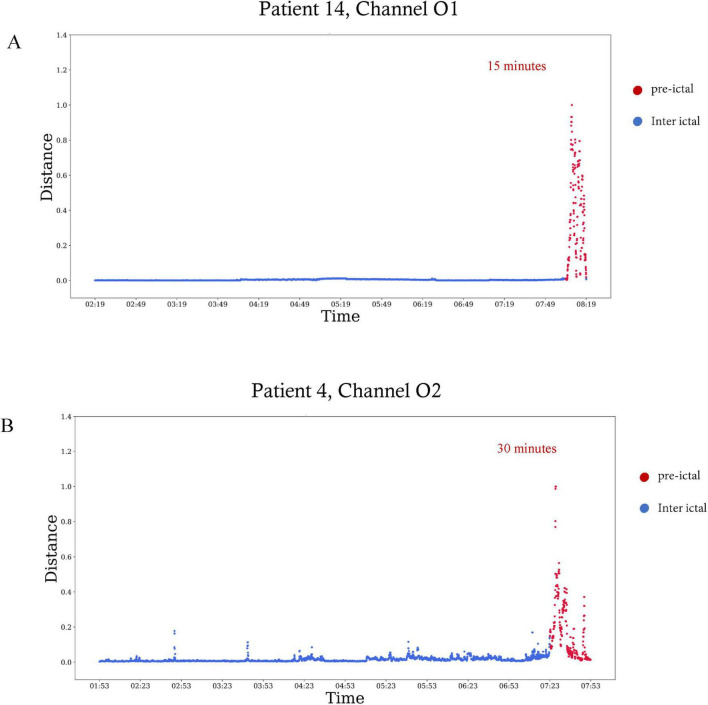
Preictal interval in EEG signals of two patients. The Mahalanobis distance as a function of time visualized over 6 h, with the plot ending at seizure onset. Red dots are included in the preictal interval. **(A)** The distance calculated for patient 14 in the EEG electrode of channel O1. The preictal interval starts 15 min before seizure onset. **(B)** The distance calculated for patient four in the EEG electrode of channel O2. The preictal interval starts 30 min before seizure onset.

Table 2 summarizes the start time and duration of the preictal interval detected for each recording of each patient in the inclusion criteria. It also shows the EEG electrode channel selected for each record, and the three features selected by the Feature-Importance algorithm. For example, the first row represents results of seizure number one of patient four from the Sienna University Hospital. The preictal interval detected started 30 min before seizure onset and its duration was 30 min, ending at seizure onset. The EEG electrode showing the best inter-ictal to preictal separation chosen by the Channel-Selection algorithm was O2. The three features selected by the Feature-Importance algorithm are Spectral Entropy, Hjorth Mobility and Delta Power. An example of preictal intervals detected for two patients is shown in Figure 3. This example also emphasizes the variability in Mahalanobis distance among patients. The algorithm failed to detect a preictal interval for two seizures of two different patients. The minimal duration measured was 15 min (as constrained by the algorithm), and the longest was 156 min. In 16 out of 23, the preictal interval ended at seizure onset. In the other seven recordings, the preictal interval ended minutes and even hours before seizure onset. On average the preictal period began 83 ± 60 min before seizure onset, its duration was on average 56 ± 47 min.

**TABLE 2:** Preictal interval detected for each recording of each patient in the inclusion criteria, the channels selected by the channel selection algorithm, and features selected by the feature importance algorithm.

Patient information		Preictal interval		Channel selected	Features selected
**Patient ID**	**Seizure ID**	**Start time before seizure onset**	**Duration**		
**Sienna University Hospital**					
4	1	30 min	30 min	O2, O1, Pz	Spectral entropy Hjorth mobility Delta power
**Rambam Health Care Campus**					
1	1	1 h 20 min	35 min	Pz	Spectral entropy Hjorth mobility Gamma power
3	1	4 h 6 min	24 min	Fpz, F8, F4	Spectral entropy Hjorth mobility Gamma power
4	1	2 h 10 min	100 min	Pz, P4, C4, F4, T4, F8, Cz, Fz, P3, C3, F3, O1, T5, Fp1	Spectral entropy Hjorth mobility Delta power
5**	1	20 min	20 min	Fpz	Spectral entropy Hjorth mobility Delta power
	2	15 min	15 min	Fpz	Spectral entropy Hjorth mobility Delta power
8	1	3 h 42 min	60 min	O2, O1, T6, F4, T3, C4, Pz, P4, P3, C3, F7, Fz	Spectral entropy Hjorth mobility Theta power
11	1	3 h	30 min	C4, F4, P3, F3, Cz, P4	Spectral entropy Hjorth mobility Delta power
12	1	24 min	24 min	F7, P3, Fz, T3, T5	Spectral entropy Hjorth mobility Theta power
13	1	1 h 18 min	18 min	F3, C4	Spectral entropy Hjorth mobility Delta power
14	1	15 min	15 min	Cz, Fpz, P4, Pz, Fz, T5, F4, F3	Spectral entropy Hjorth mobility Delta power
17*	1	45 min	45 min	P3, Cz, O2, O1, Pz, C4, P4, T5, Fpz, F8, F3	Spectral entropy Hjorth mobility Delta power
	2	1 h 30 min	20 min	P3, Cz, O1, O2, Fpz	Spectral entropy Hjorth mobility Delta power
18	1	40 min	40 min	O1, T6, P4, C3, P3	Spectral entropy Hjorth mobility Theta power
25***	1	2 h	2 h	F3, Fz, Fp1	Spectral entropy Beta power Hjorth mobility
	2	2 h 36 min	2 h 36 min	F3, Fpz, T3, Pz, Fz	Spectral entropy Theta power Hjorth mobility
26****	1	85 min	85 min	Fpz, Fp2, Fz, Cz	Spectral entropy Hjorth mobility Theta power
	2	45 min	45 min	Fpz, Fp2, O1, O2	Spectral entropy Hjorth mobility Theta power
28	1	80 min	80 min	C3, O1, O2, P4, T4, Fp2	Spectral entropy Hjorth mobility Theta power
29	1	180 min	180 min	T5, T6, T4, O1, O2, P3, F4	Hjorth mobility Gamma power Spectral entropy
31	1	180 min	180 min	T5, C3, Fp2, F4, C4, Pz	Spectral entropy Hjorth mobility Theta power
32	1	80 min	80 min	Cz, O1	Hjorth mobility Beta power Spectral entropy

*The time difference between the two seizures of patient 17 is 41 h and 9 min.

**The time difference between the two seizures of patient 5 is 15 h and 52 min.

***The time difference between the two seizures of patient 25 is above 48 h.

****The time difference between the two seizures of patient 26 is 15 h and 51 min.

### 3.2 Separation *f*eatures

**FIGURE 4:**
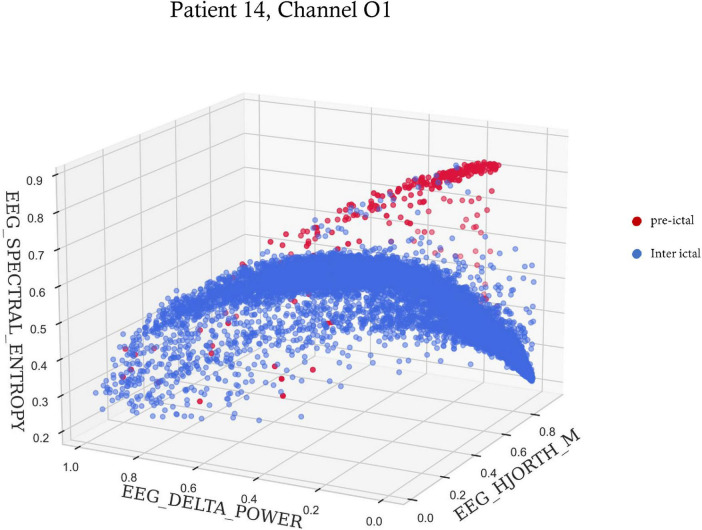
A 3-D scatter plot of three features showing preictal to interictal separation. The spectral entropy, delta power and Hjorth mobility of 6 s time windows within the 6 h preceding seizure onset for patient 14. Red dots are time points included in the preictal interval, and blue dots are time points during the rest of the time.

A summary of the three most important features selected for each seizure is shown in Table 2. Out of the 10 features, spectral entropy and Hjorth mobility, theta power, delta power, beta power and gamma power were selected among the top three most important features identified by the feature importance algorithm. Spectral entropy and Hjorth mobility were among the top three in all patients. Figure 4 shows an example of preictal to interictal separation by the three most indicative features for patient 14.

### 3.3 Channel selection

**FIGURE 5:**
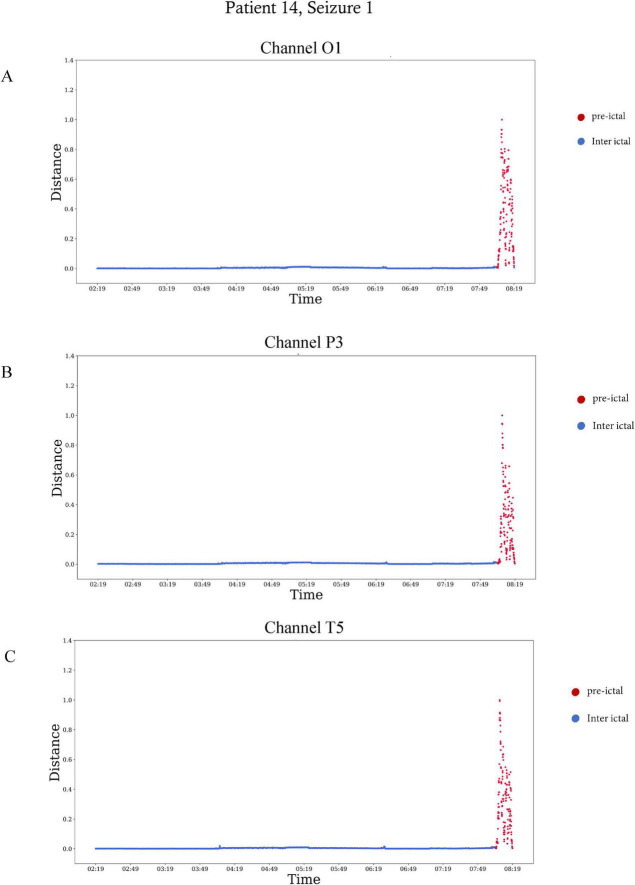
The Mahalanobis distance as a function of time in three different electrodes of patient 14, seizure number 1. The Mahalanobis distance was calculated over six the hours preceding seizure onset, from 02:19 a.m. to 08:19 a.m. The plots end at seizure onset. Red represents the preictal interval, which started 30 min before seizure onset. **(A)** The distance calculated for the EEG electrode of channel O1, **(B)** the distance calculated for the EEG electrode of channel P3, and **(C)** the distance calculated for the EEG electrode of channel T5.

**FIGURE 6:**
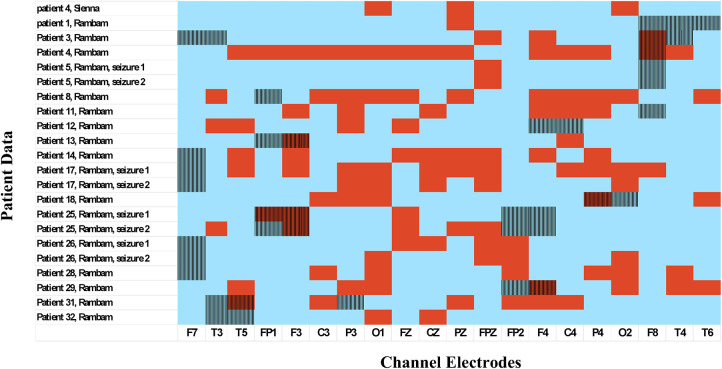
Seizure onset zone vs. preictal activity areas. EEG electrodes chosen by the channel selection algorithm as indicative of preictal activity are colored in red; electrodes not selected by the algorithm are colored in blue. Vertical lines: seizure onset zone identified by specialized epileptologists.

In Table 2, which shows the channels selected for each seizure, it can be seen that are the number of channels selected varied among patients. For example, in patient eight most channels were indicative of preictal activity, while in patient 13 only a few were indicative. This is also illustrated in Figure 5 which shows how multiple channels were indicative of preictal activity in patient 14 and the variability in the Mahalanobis distance between channels of the same patient. A comparison between the selected channels to the seizure onset zone of each seizure is illustrated in Figure 6.

### 3.4 Repeated seizures

Four patients met the criteria for including two seizures in the analysis. The time difference between the two seizures of patient 17 is 41 h and 9 min; the time difference between the two seizures of patient 5 is 15 h and 52 min, the time difference between the two seizures of patient 25 is above 48 h, the time difference between the two seizures of patient 26 is 15 h and 51 min. In all four, the localization of preictal activity was consistent for both seizures, but the start time and duration of preictal period differed between the seizures. The per-patient difference between start times of the intervals was, on average, 32 ± 16 min. The difference between the duration of preictal intervals of the same patient was, on average, 27 ± 14 min. In three out of four patients, both preictal intervals ended at seizure onset

## 4 Discussion

This work presented a novel quantitative definition of the preictal interval as prolonged intervals of highly uncommon values of features extracted from scalp EEG. Using this definition, analysis on long-term EEG recordings from 21 patients enabled investigating the duration and start time of the preictal periods and demonstrated variation between patients and between different seizures of the same patient. Feature importance analysis of the detected intervals allowed characterization of the three features best distinguishing from interictal activity, with two out of three prominent features common to all patients. Channel selection analysis enabled localization of preictal activity and which was not consistent with epileptic foci.

Previous studies set a fixed start and end time prior to seizure onset assumed to include preictal activity in all patients (Tsiouris et al., 2018; Abdelhameed and Bayoumi, 2019; Duy Truong et al., 2019; Wang et al., 2020). The unique approach presented in the current work suggests that preictal activity differs in time and duration between patients and also in the same patient.

On exploratory inspection of EEG signal features, we observed distinct intervals of uncommon brain activity preceding epileptic seizures that varied on timing and location. Based on this observation, we chose to use anomaly detection methods to find the preictal activity intervals. Detecting and analyzing those intervals, we demonstrated that preictal activity differs in its time, duration and location between patients.

In some seizures, preictal activity ends at seizure onset while, in others, it ends before seizure onset. Previous research showed that preictal activity can be detected up to hours before seizure onset (Litt et al., 2001; Bandarabadi et al., 2015). Since each individual has its own epileptic network, and the response of this network to seizure initiation varies among patients- the time until network organization leading to a seizure differs from one patient to another. Based on this knowledge, we did not define preictal activity by a fixed onset time and duration- but rather, referred to them as variables. In some patients, preictal activity ended at seizure onset, while in others it ended minutes and even hours before seizure onset. Those findings correspond with previous work regarding seizure initiation. Engel (1996) suggested that sufficient hypersynchronous activity in extensive areas in the epileptic network eventually results in the bursting of a seizure. This corresponds to the cases where preictal activity ends at seizure onset. Another theory (Engel, 1996; Trevelyan and Schevon, 2012) proposes that inhibitory neurons prevent epileptiform activity, and when they fail, a seizure bursts. This likely explains the seizures in which preictal activity ends before seizure onset, then inhibitory activity takes place, and when it fails- ictal activity is initiated. This theory is also supported by the concept of dynamic attractors presented by Khona and Fiete (2022). In such case, the preictal activity can be thought of as a shift of attractors to a chaotic state of ictal activity.

To exclude the possibility that specific patient behaviors- such as eating or sleeping- contributed to the distinct EEG patterns attributed to preictal activity, video recordings corresponding to the identified preictal intervals were reviewed by an experienced epileptologist. No consistent behavioral activity was observed that could account for the unique EEG features. As for potential confounding effects of medication, these would be expected to manifest as significantly longer time constants, whereas cognitive influences would likely result in markedly shorter time constants.

As mentioned previously, this work demonstrated that preictal activity is patient-specific and even seizure-specific. Therefore, seizure prediction models should not focus on a specific time but rather on the unique characteristics of preictal activity. A 10-feature representation of the scalp EEG signal allowed for a clear separation between preictal and interictal intervals. By using the Feature-Importance algorithm and choosing the three most important features, this separation can even be visualized in a 3D space (Figure 4). Analysis of the entire dataset found that Spectral entropy and Hjorth mobility were among the top three features of preictal activity in all patients. This suggests that preictal activity shares some common features in all patients.

Results of the channel selection algorithm demonstrated that some EEG electrodes showed better separation between preictal and interictal activity than others. This suggests that preictal activity can be localized for each seizure. Also, the localization of preictal activity does not always correlate with the seizure onset zone and can even occur on the contralateral hemisphere, as shown in Figure 6. In addition, in most seizures preictal activity was located in multiple areas on the scalp and was not limited to a single lobe. Another important finding is that localization of preictal activity varies among patients in multiple ways. First, the EEG channels chosen by the channel selection algorithm differ among patients. Second, in some patients the electrodes are near the epileptic foci while in others they are on the contralateral hemisphere. Also, while numerous channels show distinct preictal activity in some patient, in others only a few. This significant variability can be attributed to the unique epileptic network of each individual. It also emphasizes the need for a personalized channel selection algorithm in a seizure prediction system. Examination of two different seizures of the same patient revealed that localization of preictal activity was similar; this finding was evident in four different patients. This insight might be applicative in the development of patient-specific seizure prediction systems that are adapted to the prominent locations of preictal activity of a patient.

As mentioned previously, channels selected by the channel selection algorithm do not always correspond with seizure onset zone. This can be explained by the key concept of the spatial distribution of preictal deviations. It is well established that the epileptic network exerts remote effects on structurally normal brain regions. Englot et al. (2008) demonstrated in mouse models of epilepsy that during temporal lobe seizures, dysfunction in the frontal lobe can be observed. This phenomenon is not due to direct seizure involvement of the frontal cortex but rather results from remote inhibitory network effects, ultimately leading to frontal lobe dysfunction. In other words, during a seizure characterized by rapid dynamic activity, functionally uninvolved areas can exhibit significant secondary impairments. Similarly, Wong et al. (2022) investigated the impact of interictal activity, which can persist for up to 200 ms, on cognitive function. Using stereo-electroencephalography (SEEG), he demonstrated that interictal discharges disrupt synchronization within the anterior cingulate cortex (ACC), impairing attention and concentration in children. Notably, these effects were observed regardless of the spatial origin of the interictal activity. Even when the epileptic network did not anatomically involve the ACC, functional impairment was evident, indicating that the influence of interictal discharges extends beyond the primary seizure focus. Beyond these findings, interictal dysfunction can be detected using neuroimaging and cognitive assessments. Positron emission tomography (PET) studies have revealed areas of metabolic impairment during the interictal phase. In many cases- particularly in temporal lobe epilepsy- these functionality deficient zones correspond to the scalp-defined seizure onset zone (Ponisio et al., 2021). In our study, we focus the dynamic evolution of brain activity in the preictal state. Given the widespread and remote effects of epileptic activity, it is reasonable to hypothesize that dynamic changes may be detectable in brain regions beyond the core epileptic network. While chronically dysfunctional areas tend to remain persistently impaired, it is the functionally intact regions that are more likely to exhibit progressive changes in response to the initiation of a seizure process.

**FIGURE 7:**
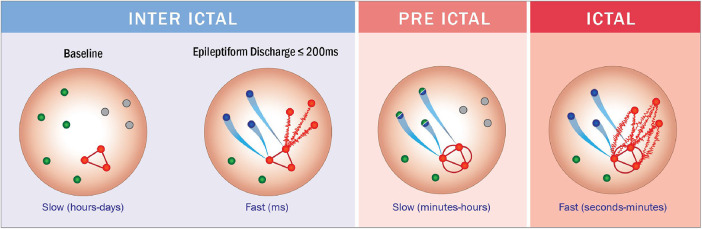
Suggested dynamic evolution of brain networks from the interictal through preictal to ictal phases. Green: Regions with normal function. Gray: Areas with chronic functional deficits. Red with solid lines: Seizure focus. Red with oscillating lines: Primary propagation zone, corresponding to the scalp-recorded interictal zone/seizure onset zone. Blue: Regions influenced by the seizure-related attractor network. Green-to-Blue Gradient: Progressive functional changes reflecting pre-seizure network reorganization. Illustrated by Sari Eran Herskovitz.

This hypothesis is consistent with the Attractor Theory (Khona and Fiete, 2022), which proposes that seizure evolution follows specific dynamical trajectories that engage brain networks beyond the ictal onset zone (Lopes Da Silva et al., 2003). Figure 7 illustrates these concepts, providing a visual representation of the key findings and the underlying hypothesis.

Results of the feature importance algorithm demonstrated that spectral entropy and Hjorth mobility are consistent across patients, while there is variation in which frequency band is most important among patients. This variation can be attributed to the unique epileptic network of each patient, as there are many configurations of seizure initiation. For example, seizures originating from cortical dysplasia are initiated by interictal activity that becomes synchronized, rhythmic and shows delta brushes. There are numerous configurations of epileptic network organization and seizure initiation- Frauscher et al. (2024), for example, discovered seven different network organization of seizure initiation, depending on epileptic foci and the underlying pathology. Therefore, patients vary from each other depending on those fundamental characteristics.

The algorithm failed to detect preictal activity for two seizures in two different patients. The failure of preictal detection is thought to be attributed to the epileptic foci in both patients. The first patient had brain surgery and her epileptic foci was discovered to be in the left supplementary frontal area. This results in a small epileptic network that is challenging to detect using scalp EEG. The second patient had anoxic brain damage since infancy with bilateral thalamocortical damage. Although her epileptic focus is in her left temporal lobe, the thalamocortical insult damages the ability to detect preictal activity in distant locations since it reduces network connectivity. The failure to detect the seizures can be also attributed to the features used- in this work we chose to investigate 10 features which are widely used in the field of neuroscience and seizure prediction, but those may not be sufficient for detection of preictal activity in some patients. Also, we used the standard scalp EEG recordings measuring 21 electrodes, which was sufficient for most patients and allowed preictal interval detection. However, using more channels could reveal changes in the epileptic network that were not detectable in the basic EEG electrodes for certain patients.

The analysis was performed on scalp EEG recordings, which represent local summations of electrical activity and large areas of the brain network. Combined with the fact that localization did not always correlate with the epileptic foci, this suggests that epileptogenesis involves a large network in areas not involved in ictal or interictal activity. It also allows examination of how normal brain areas are affected by epileptic activity. Compared to intracranial EEG, which has been widely used in recent studies, scalp EEG signals suffer from low resolution but enables implementation of non-invasive measures for seizure prediction.

It is important to emphasize that this study introduces a novel conceptual framework for analyzing the preictal period, aiming to distinguish between the stable interictal state and the preictal phase, by considering the entire brain network and recognizing that the epileptic process can have effects extending beyond the primary epileptic focus. The key innovation of this work lies in leveraging existing features rather than employing a black-box modeling approach. While further investigation is warranted, our methodology allows for reproducibility by other researchers, in contrast to many studies in this domain. Based on these findings, it may be possible to develop a simple wearable device configured to closely match the optimal electrode placement identified. However, this constitutes a separate line of research.

## 5 Conclusion

This study demonstrates that the features analyzed can uncover network dynamics that are distinct from both the interictal baseline and the ictal period. Notably, these preictal activities frequently emerged from brain regions outside the epileptogenic zone—areas not directly implicated in seizure generation but seemingly involved in broader network reorganization.

The spatial manifestation of this activity was individualized, yet recurrent across seizures within the same patient, indicating a stable patient-specific pattern. In contrast, the timing of this activity exhibited considerable variability both between patients and across different seizures in the same individual, reflecting dynamic preictal processes.

These findings provide preliminary support for the delineation of a preictal state, characterized by distinct network behavior, and suggest that seizure-related activity may extend beyond traditionally defined epileptogenic regions. Moreover, this work raises the possibility that such network dynamics could contribute to the cognitive and functional consequences often observed in epilepsy.

## 6 Limitations

This study was limited by its retrospective nature. While providing insight into the processes preceding seizure onset, further prospective research should be performed to test it in real-time. It was also limited due to its small dataset, and should be extended to larger cohort study. Furthermore, only patients evaluated by video EEG were included, which introduced a selection-bias, limiting generalizability of the conclusions to populations other than those that are drug-resistant, difficult to treat and evaluated by video EEG monitoring. In addition, results regarding multiple seizures of the same patient were derived from four patients only and are therefore highly limited. Due to the way the video-EEG monitoring was conducted, we did not examine the interictal period over 24 h but rather a fraction of the day (8–10 h). Therefore, it could be argued that the changes we observe result from diurnal variation. However, it is important to note that upon clinical review of the recordings at the identified time points, we did not find any clinical changes in the EEG. Additionally, in a significant number of patients, the period ends with a seizure. Thus, we believe our findings reflects network reorganization leading up to a seizure, but further validation is needed with additional studies and cases.

Finally, analysis was limited to seizures preceded by at least 8 h of interictal activity prior to seizure onset. Therefore, the results are limited to the first seizure among a cluster of seizures, and cannot be applied to the following seizures in a cluster.

## Data Availability

The raw data supporting the conclusions of this article will be made available by the authors, upon request.
