# Correction: Personalized preictal EEG pattern characterization: do timing and localization matter?

**DOI:** 10.3389/fnins.2025.1645680

**Published:** 2025-07-17

**Authors:** Galya Segal, Noam Keidar, Moshe Herskovitz, Yael Yaniv

**Affiliations:** ^1^Laboratory of Bioelectric and Bioenergetic Systems, Faculty of Biomedical Engineering, Technion-Israel Institute of Technology, Haifa, Israel; ^2^Faculty of Medicine, Technion-Israel Institute of Technology, Haifa, Israel; ^3^Department of Neurology, Rambam Health Care Campus, Haifa, Israel

**Keywords:** EEG, epilepsy, preictal patterns, spectral entropy, Hjorth mobility

There was a mistake in Figures 6 and 7 as published. The legend of Figure 6 is correct, but the figure itself is that of Figure 7. Similarly, in Figure 7, the legend is correct, but the figure shown is that of Figure 6. References of Figures 6 and 7 in the article are correct and should not be updated.

The original version of this article has been updated.

